# The DNA/RNA helicase DHX9 contributes to the transcriptional program of the androgen receptor in prostate cancer

**DOI:** 10.1186/s13046-022-02384-4

**Published:** 2022-05-19

**Authors:** Lidia Chellini, Marco Pieraccioli, Claudio Sette, Maria Paola Paronetto

**Affiliations:** 1 Laboratory of Cellular and Molecular Neurobiology, IRCCS Santa Lucia Foundation, Rome, Italy; 2 Department of Neuroscience, Section of Human Anatomy, Catholic University of the Sacred Heart, Rome, Italy; 3 GSTEP-Organoids Core Facility, Fondazione Policlinico Agostino Gemelli IRCCS, Rome, Italy; 4 Department of Movement, Human and Health Sciences, University of Rome “Foro Italico”, Rome, Italy

## Abstract

**Background:**

Prostate cancer (PC) is the most commonly diagnosed male malignancy and an important cause of mortality. Androgen deprivation therapy is the first line treatment but, unfortunately, a large part of patients evolves to a castration-resistant stage, for which no effective cure is currently available. The DNA/RNA helicase DHX9 is emerging as an important regulator of cellular processes that are often deregulated in cancer.

**Methods:**

To investigate whether DHX9 modulates PC cell transcriptome we performed RNA-sequencing analyses upon DHX9 silencing in the androgen-responsive cell line LNCaP. Bioinformatics and functional analyses were carried out to elucidate the mechanism of gene expression regulation by DHX9. Data from The Cancer Genome Atlas were mined to evaluate the potential role of DHX9 in PC.

**Results:**

We found that up-regulation of DHX9 correlates with advanced stage and is associated with poor prognosis of PC patients. High-throughput RNA*-*sequencing analysis revealed that depletion of DHX9 in androgen-sensitive LNCaP cells affects expression of hundreds of genes, which significantly overlap with known targets of the Androgen Receptor (AR). Notably, AR binds to the *DHX9* promoter and induces its expression, while Enzalutamide-mediated inhibition of AR activity represses *DHX9* expression. Moreover, DHX9 interacts with AR in LNCaP cells and its depletion significantly reduced the recruitment of AR to the promoter region of target genes and the ability of AR to promote their expression in response to 5α-dihydrotestosterone. Consistently, silencing of DXH9 negatively affected androgen-induced PC cell proliferation and migration.

**Conclusions:**

Collectively, our data uncover a new role of DHX9 in the control of the AR transcriptional program and establish the existence of an oncogenic DHX9/AR axis, which may represent a new druggable target to counteract PC progression.

**Supplementary Information:**

The online version contains supplementary material available at 10.1186/s13046-022-02384-4.

## Background

Prostate cancer (PC) is the most commonly diagnosed and among the main causes of cancer-related death in men [1]. Patients with localized disease, if detected at an early stage, undergo radical prostatectomy and generally display a favorable outcome. On the other hand, patients with advanced disease are addressed to chemotherapy and/or radiotherapy. Since androgen signaling plays a key role in most steps of PC pathogenesis [2], androgen-deprivation therapy (ADT) by chemical castration represents the first line treatment approach. However, most advanced PC relapse and develop resistance to ADT, resulting in castration-resistant PC (CRPC) [3]. Numerous studies have shown that androgen receptor (AR) signaling remains active to support tumor growth even at the CRPC stage [2, 4, 5]. Indeed, the mechanisms leading to acquisition of ADT resistance include mutations or amplification of the *AR* gene, expression of constitutively active AR variants, upregulation of AR coactivators and *de novo* autocrine synthesis of androgens [6]. Thus, further understanding of the mechanisms involved in the regulation of AR function in androgen-sensitive and CRPC stages might pave the ground for the development of more efficacious and long-lasting therapeutic strategies for PC patients.

DNA/RNA helicases are emerging as important regulators of many cellular processes that are often deregulated in cancer [7]. Among them, the DExH-Box helicase 9 (DHX9), also known as RNA helicase A (RHA), has been implicated in the control of genomic stability, transcription and DNA replication [8, 9]. In cancer cells, DHX9 binding to long noncoding RNAs was shown to epigenetically regulate gene expression program [10, 11] and impact mRNA stability [12, 13]. Furthermore, DHX9 was also shown to interact with several transcription factors and to modulate their transcriptional activity [14, 15]. Importantly, inhibition of the interaction of DHX9 with members of the ETS transcription factor family displayed powerful anti-tumor effects on the growth and progression of several cancer types [16–18], including PC [19–21]. Thus, while an important role for DHX9 in prostate carcinogenesis is conceivable, no direct evidence is currently available. Notably, a recent study reported that AR binds the *DHX9* promoter in renal cell carcinoma and that this event is associated with osteolytic formation and bone metastasis [22], suggesting the possibility of a crosstalk between AR and DHX9. Nevertheless, whether this functional interaction occurs in PC is currently unknown.

In this study, we set out to investigate the role of DHX9 in PC. First, we found that DHX9 is up-regulated in PC samples with respect to normal prostate tissue and its high expression is associated with worse prognosis in PC patients. Depletion of DHX9 in PC cells reduced proliferation and migration. Moreover, genome-wide transcriptome analysis revealed that a significant fraction of the DHX9-regulated genes in LNCaP cells are known targets of AR. AR and DHX9 expression are highly correlated in PC patients and AR directly induces the expression of DHX9 in PC cells. Lastly, we found that depletion of DHX9 impairs binding of AR to the promoter of some target genes and dampens androgen-induced regulation of their expression. Collectively, our results reveal that DHX9 participates in the control of the AR transcriptional program and suggest a novel molecular crosstalk between AR and DHX9 in the regulation of tumorigenic features of PC cells, which could represent a new promising therapeutic strategy for PC.

## Methods

### Cell cultures and treatment

Established human prostate cancer cells LNCaP and PC-3 were obtained from the American Type Culture Collection (ATCC, CRL-1740 and CRL-1435). LNCaP cells were maintained in RPMI-1640 medium (Lonza), supplemented with 10% Fetal Bovine Serum (FBS) (Gibco), 50 units/mL penicillin and 50 mg/mL streptomycin, 10 mM Hepes (Lonza) and 1 mM sodium pyruvate (Lonza), 1% non-essential amino acids. PC-3 cells were cultured in Dulbecco’s modified Eagle’s medium (Gibco), supplemented with 10% FBS, 50 units/mL penicillin and 50 mg/mL streptomycin. Cells were incubated at 37 °C in a humidified atmosphere with 5% CO_2_. When appropriate, LNCaP cells were incubated in *charcoal-stripped* serum (CSS) media with DHT at 10 nmol/L, or Enzalutamide (Medchem Express, 915087–33-1) at 1 μmol/L for the indicated times.

### Silencing and transient transfection

Cells were transfected with siRNAs (Sigma-Aldrich), at final concentration of 50 nM, using Lipofectamine 2000 (Thermo Fisher Scientific) according to the manufacturer’s instructions. Cells were transfected for 48 h and then lysed. siRNA sequences are:

si CTRL: 5′ GGC AGC AGA GUU CAC UGC U-dCdG.

si *DHX9*: 5′-AAG AAG UGC AAG CGA CUC UAG-dCdG.

### RNA isolation, RT − PCR and real-time quantitative PCR analyses

Total RNA was extracted from cells using Trizol Reagent (Thermo Fisher Scientific), according to the manufacturer’s instructions and 1 μg were used for retro-transcription (RT) using M-MLV Reverse Transcriptase (Promega). The RT reaction was used as template for qPCR analysis using Luna Universal qPCR Master Mix (Neb England Biolabs, #M3003) on a Quant Studio 1 real-time PCR machine. The levels of gene expression were determined by normalizing to *GAPDH* mRNA expression and expressed as relative mRNA level (2^-ΔΔct). The primers used are listed in the Supplementary Table S1.

### External datasets

**Fig. 1:**
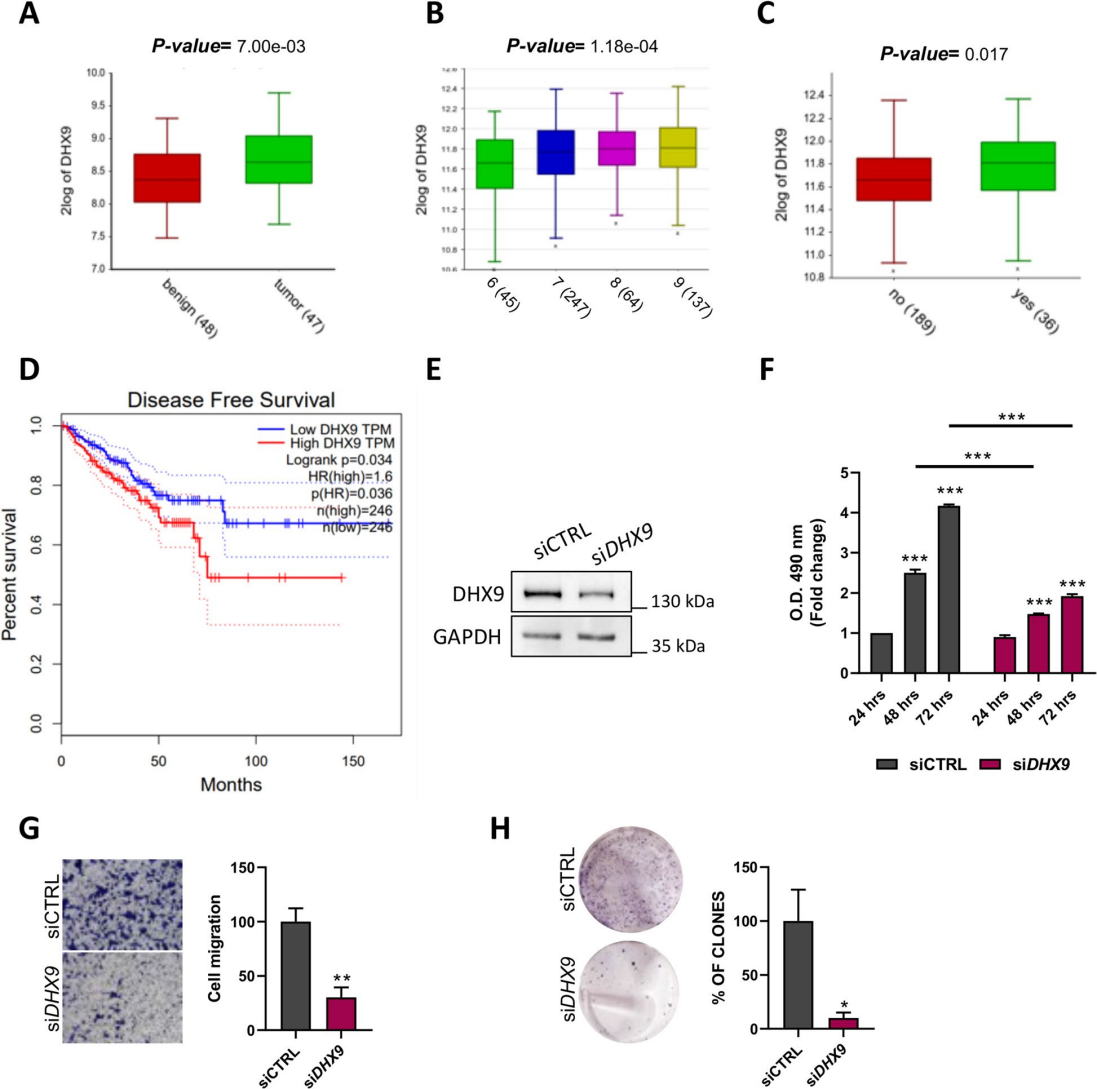
*DHX9* is associated with poor prognosis in PC patients. **A** Expression profiling of *DHX9* mRNAs in human normal and tumor specimens from Gene Expression Omnibus profile dataset (GSE29079). Statistical analysis was performed by Student’s *t* test and significance is indicated in the plot. **B** Boxplot represents *DHX9* expression in PC patients grouped according to the different Gleason score (TCGA dataset: Tumor Prostate Adenocarcinoma-TCGA-rsem-tcgars). **C** Expression profiling of *DHX9* mRNAs in human tumor specimens from stable and progressive disease, with complete or partial remission, from TCGA dataset (Tumor Prostate Adenocarcinoma-TCGA-rsem-tcgars) plotted for biochemical recurrence. Statistical analysis was performed by Student’s *t* test and significance is indicated in the plot. **D** Kaplan-Meier curve of Disease-Free Survival rate of PC patients analysed for *DHX9* expression (492 patients, TCGA dataset). The blue line shows patients with low *DHX9* expression, the red line shows patients with high *DHX9* expression. Significance is indicated in the plot. LNCaP cells were transfected with a control siRNA (siCTRL) or a siRNA specific for *DHX9* (si*DHX9*) and analyzed by WB after 48 hrs (**E**) and (**F**) MTS assay after 24, 48, and 72 hours. Values are the mean ± SD of three independent experiments, each performed in triplicate, considering the siCTRL at 24 hrs as 1. (**G**) siCTRL and si*DHX9* LNCaP cells were transfected and cultured for 48 hrs as in (**E**) and migration assay was performed. The crystal violet–stained migrating cells were photographed (*left*) and counted (*right*). Values are the mean ± SD of three independent experiments, considering the siCTRL as 100. Magnification, × 10. **H** Colony assay was performed in LNCaP cells transfected with either control (siCTRL) or *DHX9* siRNA (si*DHX9*). Histogram represents the percentage of colonies, reported as the mean ± SD of three experiments, considering the siCTRL as 100. Statistical analysis in (**F**) was performed by *two-way* ANOVA and in (**G**) and (**H**) was performed by Student’s *t* test, *p* values: *, *p* ≤ 0.05; **, *p* ≤ 0.01; ***, *p* ≤ 0.001

The patient dataset used in Fig. 1A derives from array data of 95 PCa patients (dataset GSE29079) [23]. The patient dataset used in Fig. 1B, C and D derives from array data of 492 patients (Tumor Prostate Adenocarcinoma-TCGA-rsem-tcgars) [24]. The patient dataset used for the Pearson correlation analysis in Supplementary Figs. 3A and 4C derives from array data of 370 PCa patients (GSE21034) [25]. The dataset used in Fig. 4A derives from array data of 499 PCa patients (TCGA, Firehose legacy project). The dataset used in Fig. 3C derives from RNA-sequencing experiment of LNCaP cells treated either with DMSO or enzalutamide 1 μM for 24 hours (GSE190153; Caggiano et al., under revision). The dataset used in Fig. 3B derives from Chip Seq analysis of LNCaP cells [26].

### RNA sequencing

For RNA-seq analysis, RNA from three biological replicates of either control or si*DHX9* LNCaP cells were isolated using the RNAeasy Mini Kit (Qiagen) and DNase digested, according to manufacturer’s instruction. RNA sequencing and bioinformatics analysis were performed by IGA Technology services (Via Jacopo Linussio, 51, 33,100, Udine, Italy). The quality of the reads was assessed with the software FastQC v0.11.9. The accession number for the RNA-seq data reported in this paper is GSE195916. The dataset used in Fig. 3C derives from RNA-sequencing experiment of LNCaP cells treated either with DMSO or enzalutamide 1 μM for 24 hours (GSE190153; Caggiano et al., under revision).

### Protein extraction, SDS–PAGE and Western blot analyses

For protein extract preparation, cells were washed twice with ice-cold phosphate buffered saline (PBS), resuspended in lysis buffer (50 mM Tris-HCl pH 7.5, 350 mM NaCl, 1 mM MgCl_2_, 0.5 mM EDTA, 0.1 mM EGTA, 1% Nonidet P-40, 1 mg/mL aprotinin, 1 mg/mL leupeptin, 1 mg/mL pepstatin A, 100 mg/mL PMSF). The supernatant, obtained by centrifugation at 16000 g for 15 minutes, was transferred into a new tube and used to determine protein concentrations by Bradford assay. Cell lysates were resolved by SDS/PAGE and transferred to PVDF membranes (GE, Healthcare). The membranes were blocked in TTBS (TBS with 0.1% Tween 20) containing 5% milk. Primary antibody incubations were performed in TTBS with either 5% BSA, overnight at 4 °C. After washing, the membranes were incubated with the appropriate secondary peroxidase conjugated antibody for 1 hour in TTBS. Following antibodies were used for IB: anti-DHX9 (sc-137,232), anti-GAPDH (sc-365,062), anti-AR (sc-7305), anti-H3 (Novus, NB500–171). Proteins were visualized by chemiluminescence detection system (Clarity Western ECL Substrate, #1705061) and quantification analysis was performed using Image J Software.

### Sub-cellular fractionation

Cellular pellets were re-suspended in hypotonic buffer RSB10 (10 mM Tris/HCl, pH 7.4, 10 mM NaCl, 2.5 mM MgCl_2_, 1 mM DTT, protease inhibitor cocktail). After incubation on ice for 7 min, samples were centrifuged at 1000 *g* for 7 min. Supernatant was collected as “Cytosolic extract” while pelleted nuclei were then resuspended in RSB100 (hypotonic buffer supplemented with 90 mM NaCl and 0.5% Triton X-100), sonicated, and centrifugated at 10,000 *g* for 15 min to obtain “nuclear extract”. Cytosolic and nuclear extracts were analyzed by Western Blot. For sub-cellular fractionation, LNCaP cells pellets were washed with PBS/1 mM EDTA, gently centrifuged and resuspended in ice-cold NP-40 lysis buffer (10 nM Tris-HCl pH 7.5, 0,15% NP-40, 150 mM NaCl, protease inhibitor cocktail (Sigma-Aldrich), 1 mM dithiothreitol, 0,5 mM Na-orthovanadate) for 5 min. Lysates were layered on 2,5 vol of chilled solution of 24% sucrose lysis buffer and centrifuged 10 min at 14000 rpm. The supernatant (cytoplasmic fraction) was collected and pellets (nuclei) were washed with PBS/EDTA 1 mM, gently centrifuged and resuspended in a chilled glycerol buffer (20 mM Tris-HCl pH 7.9, 75 mM NaCl, 0,5 mM EDTA, 0,85 mM dithiothreitol, 0,125 mM PMSF and 50% Glycerol). An equal volume of cold nuclei lysis buffer (10 mM Hepes pH 7.6, 7,5 mM MgCl2, 0,2 mM EDTA, 0,3 M NaCl, 1 M UREA, 1% NP-40 protease inhibitor cocktail (Sigma-Aldrich), 1 mM dithiothreitol, 0,5 mM Na-ortovanadate) was added. Tubes were vortexed twice for 2 s and centrifuged for 2 min at 14000 rpm. The supernatant was collected as soluble nuclear fraction. The chromatin pellet was washed with cold 1 × PBS/1 mM EDTA, sonicated and then centrifugated for 2 min at 14000 rpm.

### Immunoprecipitation

Precleared whole-cell lysates were incubated with anti-AR antibody or with anti-rabbit IgG Ab (Thermo Fisher Scientific) and protein G agarose beads (Thermo Fisher Scientific) at 4 °C overnight. Isolated complexes were analyzed by Western blot analysis, as previously described [27].

### Cell viability

MTS assay was performed to assess LNCaP cellular viability. Briefly, 5x10^3^ cells were plated in each well of a 96-culture plate. The absorbance (O.D. 490 nM) was measured after 24, 48 and 72 hours, by using Cell Titer Aqueous Assay (Promega) with MTS tetrazolium, following manufacturer’s instructions.

### Migration assay

1 × 10^5^ cells cells were left to migrate for 12 h at 37 °C. The migrated cells were fixed with 70% ethanol, at room temperature for 10 minutes. Next, transwell membranes were stained with 0.2% Crystal Violet solution (Sigma-Aldrich) for 7 minutes. Membranes were dipped into distilled water to remove the excess crystal violet and allowed to dry. The experiment was performed in triplicates for all conditions described. From every transwell, four images were taken at × 10 magnification. Quantification of the individual photos were performed using Image J Software.

### Clonogenic assay

For clonogenic assay, single-cell suspensions were plated in 35 mm plates at low density (2000 cells/plate). After 10 days, cells were fixed in methanol for 10 minutes and stained overnight with 0.01% Crystal Violet solution. Plates were then washed twice with water and dried. Pictures were taken to count the colonies. Results represent the mean ± SD of three experiments.

### Chromatin immunoprecipitation

LNCaP cells were cross-linked with the addition of 1% (vol/vol) formaldehyde (Sigma Aldrich) to the culture medium for 15 min at room temperature and then quenched in 125 mM glycine (Sigma Aldrich) for 5 min. Cells were washed in cold PBS and lysed in nuclei extraction buffer, containing 5 mM PIPES (pH 8.0), 85 mM KCl, NP40 0.5%, 1 mM dithiothreitol, 10 mM β-glycerophosphate, 0.5 mM Na_3_VO_4_, and protease inhibitor cocktail (Sigma-Aldrich). Isolated nuclei were lysed in a buffer containing 1% SDS, 10 mM EDTA, and 50 mM Tris-HCl (pH 8.0), 1 mM dithiothreitol, 10 mM β-glycerophosphate, 0.5 mM Na_3_VO_4_, and protease inhibitor cocktail (Sigma-Aldrich) and sonicated with Bioruptor (Dyagenode) 2 × 6 min (30 sec sonication and 30 sec pause). Chromatin extracts containing DNA fragments were pre-cleared 2 hours on Protein A/agarose/salmon sperm DNA (Millipore) and then immunoprecipitated overnight using 2 μg of anti-AR (sc-7305) or Rabbit IgG (Sigma-Aldrich). Immunoprecipitated DNA was recovered according to standard procedures and analyzed by qPCRs, using primers listed in Supplementary Table S1. DNA associated with AR is represented as percentage of input.

### Bioinformatic analysis

The Disease-Free Survival (DFS) analyses of TCGA data was performed using GEPIA (Gene Expression Profiling Interactive Analysis) online tool [28].

GSEA analysis was performed using the fgsea package [29] in the R environment using the Hallmark gene sets (v.7.5.1) obtained from https://www.gsea-msigdb.org. *P*-value adjusted < 0.05 value was applied to sort and select enriched gene sets. GSEA plot was obtained using an in-house script in the R environment.

The RNA-Seq libraries were sequenced on paired-end 150 bp mode on NovaSeq 6000. The raw paired-end reads were preprocessed to mask adapters with cutadapt (v.1.11) [30] and corrected to remove lower quality bases and adapters with ERNE (v.2.1.1) [31]. The reads were then mapped to the human genome (hg38) with iGenomes gene annotation using STAR aligner (v.2.6.1d) [32]. Statistics on strandness reads, genebody coverage, reads distribution and insert size estimation were performed using RSeQC (v.4.0.0) [33]. The uniquely mapped reads from biological replicates were kept and counted with HTSeq (v.0.11.1) [34]. Based on these read counts, normalization and differential gene expression were performed using DESeq2 (v.1.30.0) [35] by fitting a Generalized Linear Model (GLM) for each gene. Statistical significance was determined using a Wald test.

### Gene ontology

Functional gene annotation clustering for si*DHX9* regulated genes was performed by using DAVID Bioinformatic Database (https://david.ncifcrf.gov/summary.jsp).

### Statistical analysis

Statistical analyses were performed with the GraphPad Prism (GraphPad Software) and the values represent mean ± SD obtained with not less than three independent experiments. The statistical significance of the differences was determined by the Student’s *t* test or *two-way* ANOVA test.

## Results

### DHX9 is highly expressed in prostate cancer patients and contributes to the tumorigenic phenotype

Analysis of a public dataset reporting expression data from PC (*n* = 47) and benign prostate (*n* = 48) samples (GSE29079) indicated that *DHX9* expression levels were significantly higher (*p value* **=** 7.00 e^− 03^) in PC samples compared to non-cancer samples (Fig. 1A). Moreover, analysis of RNA-seq data from The Cancer Genome Atlas (TCGA; Tumor Prostate Adenocarcinoma-TCGA-rsem-tcgars) revealed that increased *DHX9* expression significantly correlated with PC stage progression, as measured by Gleason score (Fig. 1B). Moreover, DHX9 expression was evaluated in human tumor specimens from stable and progressive disease, with complete or partial remission, from the same TCGA dataset, undergoing or not biochemical recurrence. This analysis showed that DHX9 expression is significantly increased in recurrent patients (*p value* **=** 0.017; Fig. 1C). Kaplan-Meier curves also indicated that high *DHX9* expression was significantly associated with shorter disease-free survival (DFS) in PC patients (*p* = 0.034; Fig. 1D). These data reveal a positive correlation between DHX9 expression, PC tumorigenesis and patient’s clinical outcome, hence suggesting the clinical relevance of DHX9 function in prostate carcinogenesis.

Most PCs are androgen-sensitive and maintain a high response rate to androgen deprivation therapy (ADT) [5]. Thus, to investigate the role played by DHX9 in PC, we employed the androgen-sensitive LNCaP cell line, a well-established *in vitro* model for the study of androgen-mediated PC tumorigenesis [36, 37]. Knockdown of DHX9 by transient transfection of LNCaP cells with specific siRNAs showed a significant reduction of the proliferation rate after 48 hours, which was sustained for at least 72 hours (Fig. 1E, F). In addition, DHX9-depleted LNCaP cells exhibited strongly decreased migration capacity (Fig. 1G) and clonogenic potential (Fig. 1H). The same analysis was also performed in the metastatic androgen-insensitive PC-3 cells. Like in LNCaP cells, DHX9 depletion (Suppl. Fig. 1A, B) affected cell proliferation and migration of PC-3 cells (Suppl. Fig. 1C, D), albeit at lower extent than in the AR-sensitive cells.

Taken together, these results suggest that DHX9 plays an important role in PC by supporting oncogenic features associated with the tumor phenotype.

### DHX9 controls the expression of tumorigenic-related genes in LNCaP cells

**Fig. 2:**
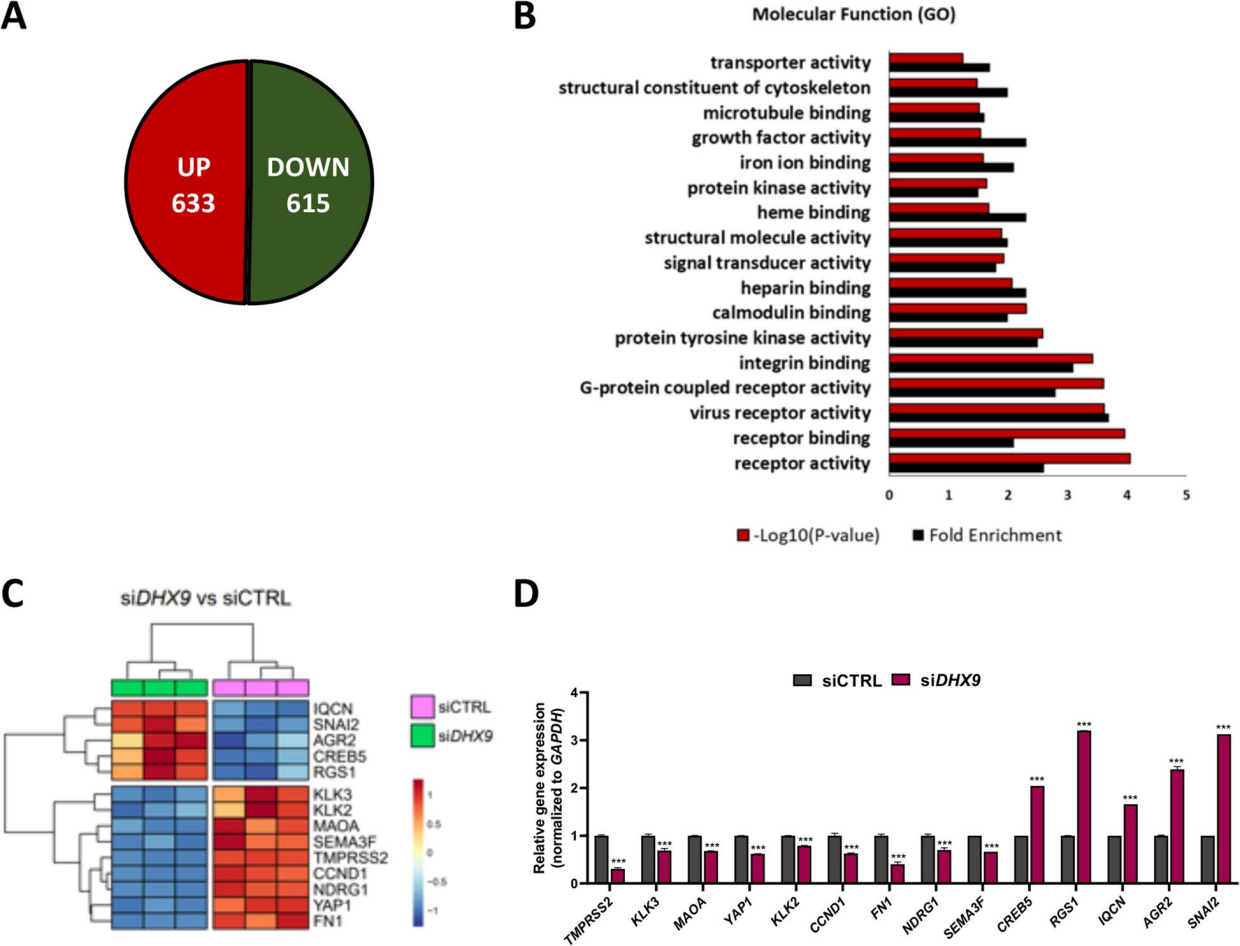
*DHX9* depletion affects gene expression in LNCaP cells. **A** Schematic representation of the distribution of up and down regulated genes upon DHX9 silencing. **B** Gene Ontology (performed using DAVID) of terms regulated at gene expression levels by analyzing DEGs after *DHX9* silencing. Histograms represent the Fold Enrichment score and the -log_10_ (*p-*values). **C** Heatmap summarizing the outcome of the RNA sequencing for the genes selected for RT-qPCR validations. **D** Validation of DEGs regulated by *DHX9* silencing was performed by RT-qPCR analyses, using the indicated primers. Values are the mean ± SD of three independent experiments, considering the siCTRL as 1. Statistical analyses were performed by Student’s *t* test, *p* values: ***, *p* ≤ 0.001

DHX9 contributes to several layers of gene expression regulation, including transcriptional activation and RNA processing [9, 14]. To elucidate the mechanism underlying the impact of DHX9 on PC cell biology, we performed transcriptomic analysis of LNCaP cells in which *DHX9* expression was knocked down by RNA interference (Suppl. Fig. 2A, B). Depletion of DHX9 protein (Suppl. Fig. 2C) caused extensive transcriptional changes in LNCaP cells, with 1248 genes that were differentially expressed (DEGs; fold change > 1.5, *p* < 0.05; Suppl. Table S2). DEGs were equally distributed between up- (*n* = 633) and down-regulated (*n* = 615) (Fig. 2A). Gene ontology (GO) analysis of the DEGs using the DAVID database indicated that processes like “cell adhesion”, “signal transduction”, “regulation of cell proliferation” and “cell migration” were significantly enriched (Suppl. Fig. 2D), providing support to the effects elicited by *DHX9* knockdown in LNCaP cells and to the involvement of DHX9 in the control of prostate carcinogenesis. In addition, DEGs were significantly enriched in molecular functions related to “receptor binding” and “receptor activity” (Fig. 2B), especially among the down-regulated genes, indicating that DHX9 is required for the expression of these genes in PC cells (Suppl. Fig. 2E, F). Validation of the expression changes of 14 arbitrarily selected genes by quantitative real time PCR (qPCR) using an independent set of LNCaP samples confirmed the reliability of the RNA-seq and bioinformatics analyses (Fig. 2C, D).

### *DHX9* silencing affects the expression of known AR targets

**Fig. 3:**
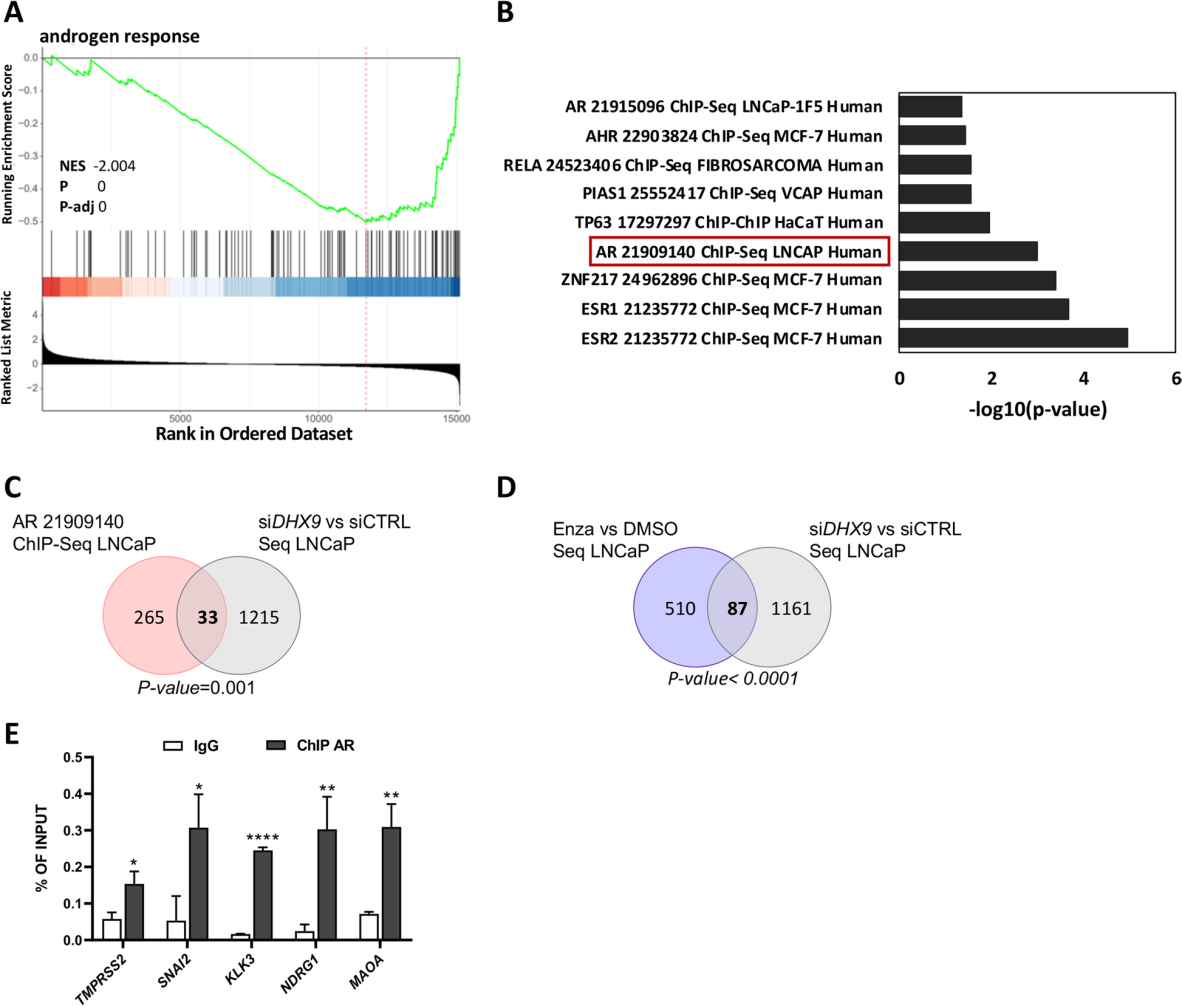
DEGs upon *DHX9* silencing are enriched in AR targets. **A** Gene Set Enrichment analysis (GSEA) of androgen response gene signature in siDHX9 versus siCTRL LNCaP cells. Androgen response resulted as the second most significant category after mTORC signaling (Suppl. Table S3). Bars represent individual genes in a ranked data set list. NES, normalized enrichment score. **B** Gene set enrichment analysis (performed via EnrichR) of DEGs after si*DHX9.* The overlap between the two analysis is shown in the Venn diagram (*p-value* = 0.001) in (**C**). **D** DEGs after *DHX9* silencing were overlapped with DEGs in response to Enzalutamide treatment (*p-value* < 0.0001). **E** qPCR analyses of ChIP experiments performed in LNCaP cells using AR antibody and anti-IgG rabbit Abs (IgG). Associated DNA was expressed as % of input. Values are the mean ± SD of three independent experiments. Statistical analysis was performed by Student’s *t* test, *p* values: *, *p* ≤ 0.05; **, *p* ≤ 0.01; ****, *p* ≤ 0.0001

Since DHX9 modulates the expression of genes related to receptor functions and the AR-driven transcriptional program is fundamental for PC tumorigenesis [4, 5], we asked whether DHX9 may contribute to the AR-driven transcriptional program in PC cells. Interestingly, Gene Set Enrichment Analysis (GSEA) using HALLMARK highlighted Androgen Response as significantly enriched among the DHX9 DEGs (Fig. 3A and Suppl. Table S3). Moreover, by querying the chromatin immunoprecipitation (ChIP) Enrichment Analysis (ChEA) database with the EnrichR tool (https://maayanlab.cloud/Enrichr/), we found that DHX9-modulated genes are significantly enriched in known AR targets in LNCaP cells [26] (Fig. 3B). In particular, this analysis identified 33 genes that were both modulated after *DHX9* silencing and displayed AR ChIP-Seq signals in their promoter region (*p* = 0.001; Fig. 3C), suggesting that DHX9 modulates the expression of a significant fraction (> 10%) of the AR target genes.

In line with a role for DHX9 in the AR signaling pathway, we also found a significant overlap (*p* < 0.0001) between genes modulated by *DHX9* silencing and by enzalutamide-mediated AR inhibition in the same PC cells (GSE190153; Fig. 3D). To experimentally validate these computational analyses, we performed AR ChIP assays in LNCaP cells. Among the commonly-regulated genes, we verified the recruitment of AR on the promoters of Transmembrane Serine Protease 2 (*TMPRSS2*), Snail Family Transcriptional Repressor 2 (*SNAI2)*, Kallikrein 3 (*KLK3)*, Monoamine Oxidase A gene (*MAOA)* and N-myc Downstream Regulated Gene 1 (*NDRG1)* (Fig. 3E), which are all known AR target genes [38–40]. These data strongly suggest a yet unexploited connection between DHX9 and AR in PC.

### AR modulates *DHX9* expression in LNCaP cells

**Fig. 4:**
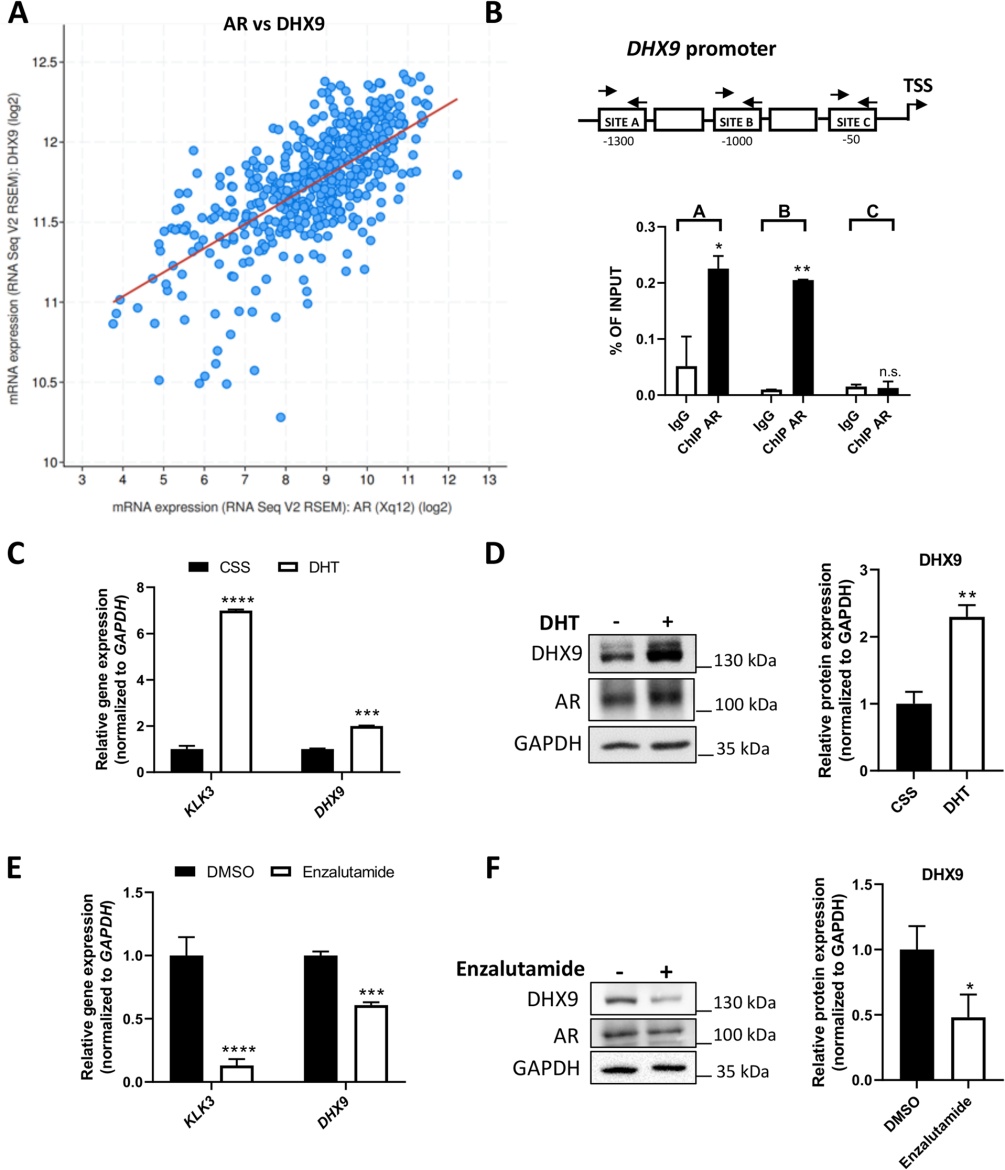
AR binds to the *DHX9* promoter and modulates its expression. **A** The plot shows the Pearson correlation between *AR* and *DHX9* expression in 499 patients with PC (TCGA, Firehose legacy project). **B** qPCR analyses of ChIP experiments performed in LNCaP cells using AR antibody and anti-IgG rabbit Abs (IgG). Associated DNA was expressed as % of input. The sequences tested for the AR binding on the *DHX9* promoter are indicated. LNCaP cells were cultured in *CSS* for 48 hrs, stimulated with DHT (10 nM) for 24 hrs and the expression of *DHX9* was measured by qPCR (**C**) or WB analysis (**D**). Quantification of mRNA and protein level are shown in the bar graphs as the mean ± SD of three independent experiments, considering the control sample (*CSS*) as 1. LNCaP cells were treated with Enzalutamide for 24 hours (1 μM) and DHX9 expression was measured by qPCR (**E**) or WB analysis (**F**). Histogram represents the mean ± SD of three independent experiments, considering the DMSO sample as 1. Statistical analyses were performed by Student’s *t* test, *p* values: *, *p* ≤ 0.05; **, *p* ≤ 0.01; ***, *p* ≤ 0.001; ****, *p* ≤ 0.0001

To further explore the functional connection between DHX9 and AR, we first analyzed their expression in primary PC samples. Analysis of gene expression data from 499 PCa patients (TCGA, Firehose legacy project) revealed a highly significant positive Pearson’s correlation (*p* = 4.68e^− 66^) between AR and DHX9 expression (Fig. 4A). A similar positive correlation was also observed in another cohort of 370 PC patients [25] (*p* = 4.19e^− 13^; Suppl. Fig. 3A). These analyses suggested that DHX9 expression might be under the control of AR in PC cells. To directly test this hypothesis, we performed AR ChIP assays in LNCaP cells. By querying the Eukaryote Promoter Database (EPD) (https://epd.epfl.ch//index.php), we identified three putative AR binding sites on the *DHX9* promoter, which we named “site A”, “site B” and “site C” from the distal-most to the proximal-most one with respect to the transcription start site (TSS). PCR analysis of the DNA that was specifically immunoprecipitated with AR confirmed its specific recruitment to the two predicted distal sites (A and B) in the *DHX9* promoter, but not to the proximal site C (Fig. 4B).

Next, to test whether AR can promote DHX9 expression, we induced AR transactivation by treating LNCaP cells with 5α-dihydrotestosterone (DHT), which binds AR with high affinity [4]. As expected, DHT strongly induced the expression of the *KLK3* gene (encoding the Prostate Serum Antigen, PSA) in LNCaP cells (Fig. 4C). Moreover, we observed that DHT induces an ~ 2-fold increase in DHX9 expression at both RNA and protein level (Fig. 4C, D). Coherently with this result, androgen deprivation (Suppl. Fig. 3B, C) or treatment with the AR antagonist Enzalutamide (Fig. 4E, F) significantly repressed DHX9 expression at both RNA and protein level. These results indicate that *DHX9* is an androgen responsive-AR target gene in PC cells.

### DHX9 interacts with AR, promotes its recruitment on gene promoters and contributes to AR transcriptional activity

**Fig. 5:**
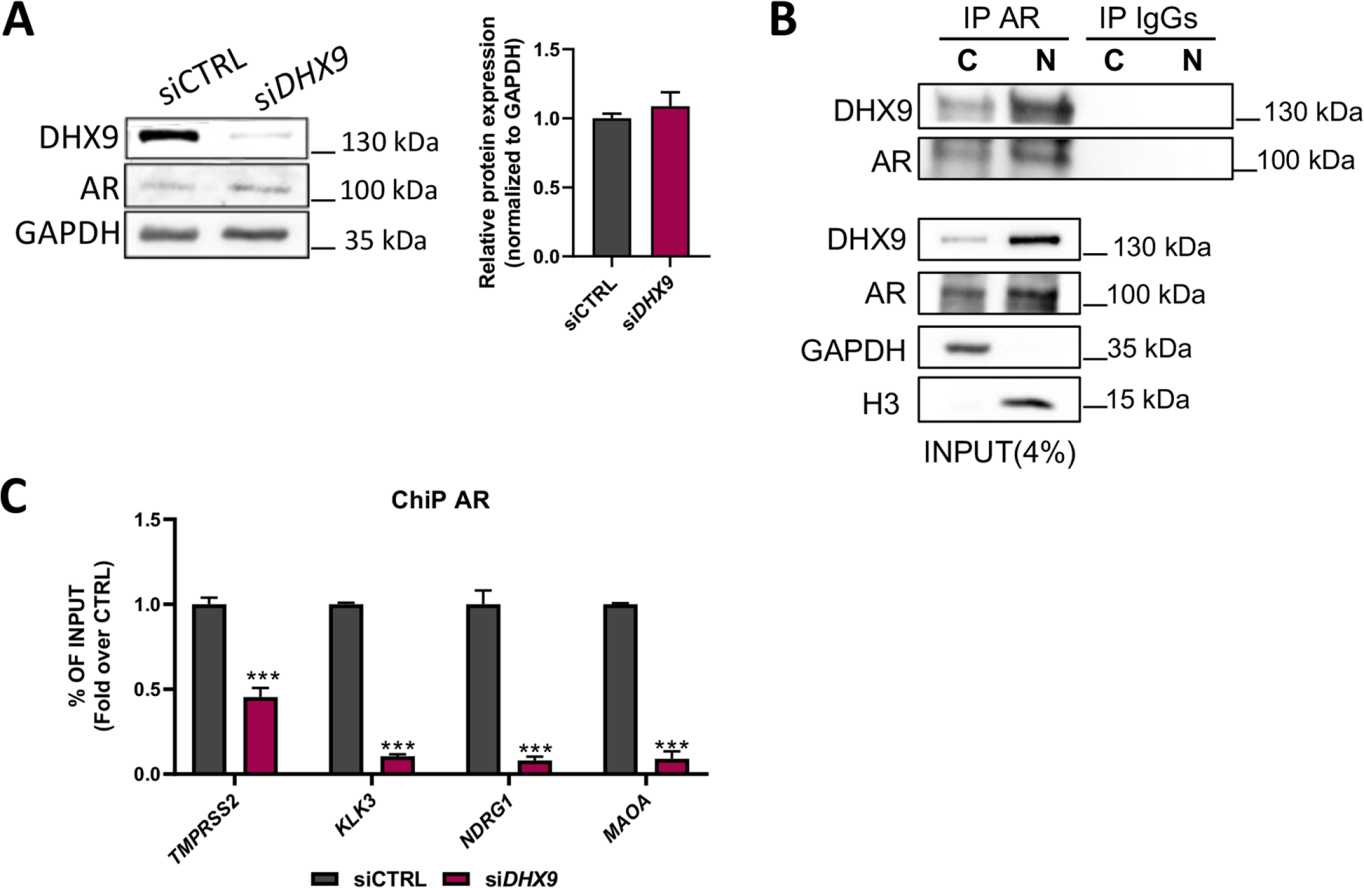
DHX9 interacts with AR and is required for the AR recruitment on the promoter of its target genes. **A** LNCaP cells were transfected with either control siRNA (siCTRL) or a siRNA specific for *DHX9* (si*DHX9*) for 48 hrs. DHX9 and AR expression was measured by WB analysis. Histogram represents the relative AR protein expression versus siCTRL, normalized to GAPDH expression. **B** Cytosolic (C) and nuclear (N) LNCaP extracts were immunoprecipitated with AR antibody (IP AR). IP and input were subjected to WB to analyse the expression of DHX9 and AR. GAPDH and H3 were used as cytosolic and nuclear markers*.* **C** LNCaP cells were transfected with a control siRNA (siCTRL) or a siRNA specific for *DHX9* (si*DHX9*) for 48 hours and ChIP experiments were performed using AR antibody. Associated DNA was measured by qPCR and histogram represents the fold over CTRL, expressed as % of input. Statistical analysis was performed by Student’s *t* test, *p* values: ***, *p* ≤ 0.001

*DHX9* silencing modulates the expression of several AR target genes, without influencing AR expression (Fig. 5A), suggesting a direct implication of DHX9 in AR signaling. To test whether DHX9 and AR are involved in a functional interaction, we first analyzed their cellular localization in LNCaP. Western blot analyses showed that both proteins are prevalently localized in the nucleus (Fig. 5B) and immunoprecipitation experiments indicated a physical interaction between DHX9 and AR both in the cytoplasm and in the nucleus (Fig. 5B). The interaction of DHX9 with AR was not dependent on RNA, as it was resistant to RNAse treatment (Suppl. Fig. 4A). Moreover, DHT stimulation of LNCaP cells that were deprived of androgens for 48 hours, promoted translocation of both AR and DHX9 in the nuclear compartment and their association with the chromatin (Suppl. Fig. 4B), suggesting that these proteins could interact in a functional complex.

To test whether DHX9 directly contributes to the transcriptional activity of AR, we performed ChIP assays on the promoter of genes that were co-regulated by the two proteins. Interestingly, depletion of DHX9, after 48 hours, strongly impaired the recruitment of the AR on the promoter region of the *TMPRSS2*, *KLK3*, *NDRG1*, *MAOA* genes (Fig. 5C). Coherently with this regulation, analysis of expression data from 370 PC patients (GSE21034) highlighted a significant positive correlation between DHX9 and these AR target genes (Suppl. Fig. 4C).

**Fig. 6:**
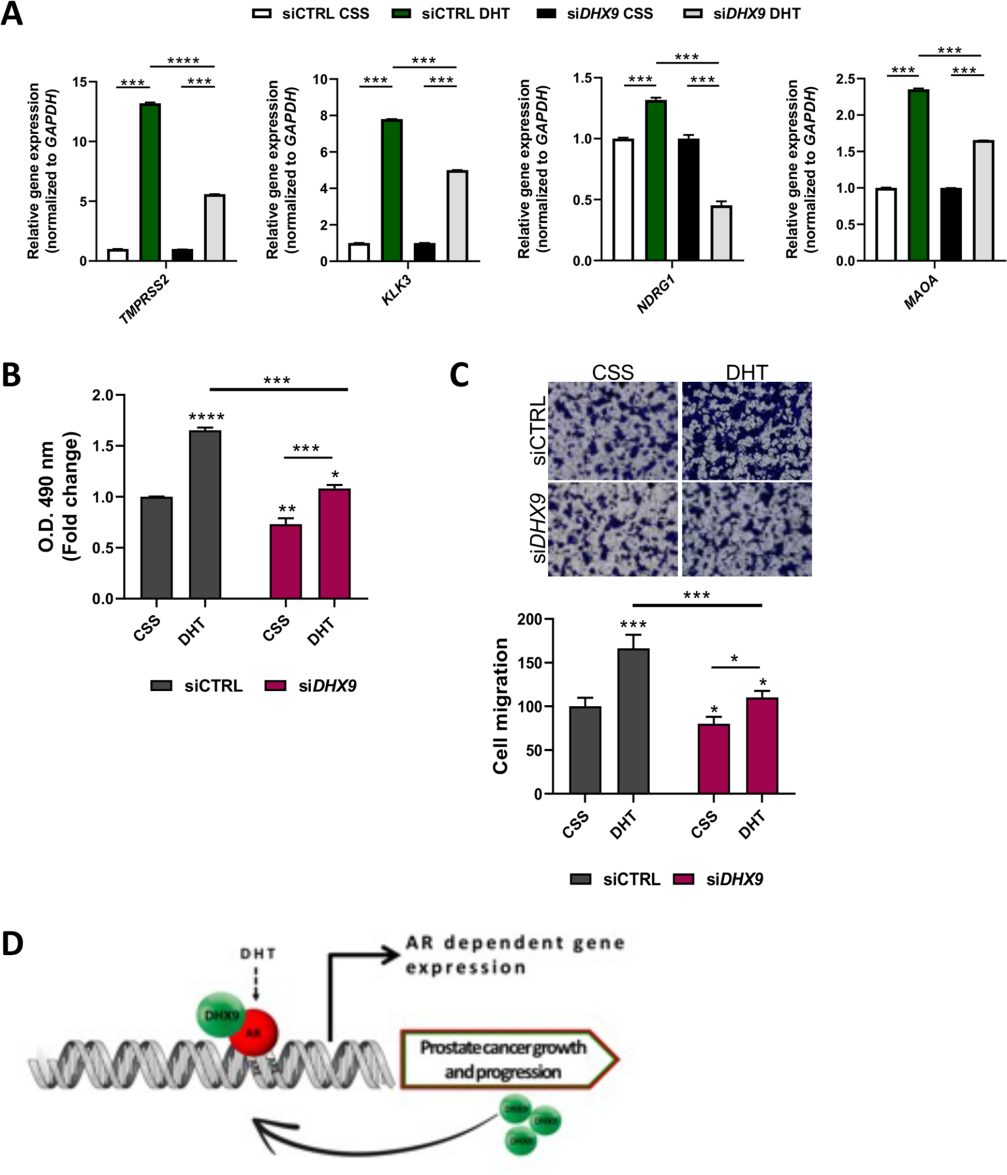
DHX9 contributes to the androgen-mediated AR program. **A** LNCaP cells were transfected with a control siRNA (siCTRL) or a siRNA specific for *DHX9* (si*DHX9*), cultured for 48 hrs in CSS, and treated or not with DHT (10 nM). qPCR was performed to analysed the expression level of the indicated genes. Histogram represents the mean ± SD of three independent experiments, expressed as fold change, considering the CSS samples as 1. **B** LNCaP cells were transfected, cultured as in (**A**) and analyzed by MTS assay after 48 hrs. Values are the mean ± SD of two independent experiments, each performed in triplicate, considering the siCTRL CSS samples as 1. **C** LNCaP cells were transfected and cultured for 48 hours as in (**A**) and migration assay was performed. The crystal violet–stained migrating cells were photographed (*upper*) and counted (*bottom*). Values are the mean ± SD of three independent experiments, considering the CSS samples as 100. Magnification, × 10. Statistical analysis in (**A**), (**B**) and (**C**) were performed by Student’s *t* test, *p* values: *, *p* ≤ 0.05; **, *p* ≤ 0.01; ***, *p* ≤ 0.001; ****, *p* ≤ 0.0001. **D** Proposed model of the DHX9/AR crosstalk

Since AR regulates the expression of its target genes in response to androgens [41], we tested whether DHX9 participates to this process. As expected, DHT stimulation induced the expression of *TMPRSS2*, *KLK3*, *NDRG1* and *MAOA* in LNCaP cells transfected with a control siRNA (si-CTRL). Notably, although it did not completely abolish it, depletion of *DHX9* significantly reduced the effect of DHT on transcription of these AR target genes (Fig. 6A, Suppl. Fig. 4D). These results further support the existence of a functional interaction between AR and DHX9 in PC cells.

Since androgens stimulate PC cell growth and migration [42], we next investigated the involvement of DHX9 in these DHT-mediated cellular events. Knockdown of DHX9 expression significantly reduced DHT-induced proliferation (Fig. 6B) and migration (Fig. 6C) of LNCaP cells. Collectively, these results indicate that AR induces the expression of DHX9, which in turn interacts with AR, promotes its recruitment to the promoter of target genes and enhances its transcriptional activity and biological function in response to androgen stimulation of PC cells (Fig. 6D).

## Discussion

PC onset and progression is driven by the transcriptional activity of the AR, a member of the nuclear hormone receptor family of transcriptional factors [43]. Ablation of androgens represents the first line therapy in the early stages of the disease. However, PCs often progress to a CRPC for which no effective cure is currently available [2, 5]. Several AR transcriptional coregulators have been identified, and some of them were shown to play critical roles in PC progression [44]. Nevertheless, current therapies are not directed at suppressing these functional interactions, possibly due to the lack of specific inhibitors. In this study, we reveal a novel role for the RNA/DNA helicase DHX9 as an AR coactivator in PC, which promotes PC cell proliferation and migration in response to androgen stimulation. Noteworthy, inhibitors of the interaction between DHX9 and other transcription factors, such as members of the ETS family, have been developed and have shown promising preclinical results in various tumors [16–18, 45, 46], including PC [19, 20, 47, 48]. Thus, our study uncovers a new oncogenic axis composed by AR and DHX9 which can be possibly exploited therapeutically.

Several helicases have been involved in the AR-dependent transcriptional regulation. Recently, a mutagenesis screen in yeast for mutants incapable of supporting the nuclear export signal (NES) of the AR, identified Prp43 as a potential factor regulating AR subcellular localization and function [49]. In this screen the nuclear localization signal (NLS) of AR fused to the GFP displayed a marked nuclear localization in the yeast model [50], whereas fusion with both NLS and NES was cytoplasmic. Notably, Prp43 mutants did not support cytoplasmic localization [49]. DHX15, the mammalian ortholog of Prp43, is a member of the DEAH-box (DHX) RNA helicase family that acts as an AR coactivator in PC cells [49]. DHX15 knockdown in PC cells reduced the expression of AR target genes, including *KLK3* (PSA), *TMPRSS*2, *NKX3.1* and *SCL45A3*, indicating an important role for DHX15 in regulating the expression of a subset of AR downstream genes [49]. Notably, DHX15 was shown to be upregulated in PC and its expression was highly correlated with Gleason scores and PSA recurrence [49, 51]. Furthermore, DHX15 knockdown reduced AR sensitivity to DHT and inhibited cell growth at low DHT levels, enhancing enzalutamide inhibition of growth in the androgen-resistant C4–2 cell line [51]. These observations suggest that DHX15 upregulation in PC could contribute to cancer progression and castration resistance. Likewise, the p68 DEAD box RNA helicase 5 (DDX5), which is a growth- and developmentally- regulated prototypic member of the DDX family [15, 52] and functions as an AR co-activator, is significantly upregulated in PC, and it was suggested to play a role in the progression to hormone-refractory stages [53]. Furthermore, the helicase DDX39B helicase, which is essential for the U2 snRNP-branch point interaction [54], was identified as a regulator of the expression of the constitutively active AR-V7 splice variant [55], which lacks the ligand binding domain and contributes to acquisition of ADT resistance [56]. Our study now provides evidence that DHX9 is an additional helicase involved in PC tumorigenesis and in the AR guided transcriptional network.

DHX9 is upregulated and plays key roles in several human tumors [9, 57–60]. Noteworthy, although the *DHX9* gene was mapped in the major susceptibility *locus* for PC (chromosome band 1q25) more than 20 years ago [61], the possible role of DHX9 in PC cells and its contribution to PC tumorigenesis has remained completely unknown to date. Our work now demonstrates that DHX9 is significantly upregulated in PC with respect to normal prostate tissue and its high expression correlates with advanced PC stages and worse prognosis in PC patients. Genome wide transcriptome analysis revealed hundreds of genes whose expression is susceptible to DHX9 levels in PC cells. Among them, we have identified several well-known AR target genes, such as *KLK3* and *TMPRSS2*, and analyses of deposited ChIP-seq studies revealed a significant overlap between genes that are bound by AR at the promoter level and those regulated by DHX9 in LNCaP cells. In further support of a functional interaction between AR and DHX9, we found that AR promotes DHX9 expression at both mRNA and protein levels in PC cells. This regulation is likely direct, as AR is specifically recruited to the *DHX9* promoter in LNCaP cells, as previously reported in renal carcinoma cells [22], while AR inhibition under androgen deprivation or enzalutamide treatment caused repression of DHX9 expression. In turn, DHX9 physically interacts with AR and enhances its ability to bind the promoters and to stimulate the expression of a fraction of its target genes. Notably, depletion of DHX9 reduced androgen-dependent proliferation and migration of PC cells, two processes that are stimulated by activation of AR. These findings support a role for DHX9 in a positive feedback loop that enhances AR transcriptional activity and function in PC cells.

DHX9 is also known to interact with other oncogenic transcription factors and to modulate their activity. For instance, DHX9 cooperates with the oncogenic transcription factor EWS-FLI1 in Ewing sarcoma to promote oncogenic transformation [62]. DHX9 depletion or inhibition of EWS-FLI1/DHX9 interaction inhibited Ewing sarcoma cell growth and improved sensitivity to therapies [8, 16]. DHX9 binds to and resolves mutagenic intra-molecular triplex structures through its helicase activity [63, 64], thus preventing genomic instability and assisting the maintenance of DNA integrity in the replication, recombination, and repair processes, also by recruiting BRCA1 to damage sites [65]. Cells that are deficient in DHX9 are impaired in the recruitment of RPA and RAD51 to sites of DNA damage and fail to repair double strand breaks (DSB) by homologous recombination [65]. Consequently, these cells are hypersensitive to treatment with genotoxic agents such as camptothecin and Olaparib [65], etoposide and UV irradiation [8], which block transcription and generate DSBs.

Inhibition of the EWS-FLI1/DHX9 interaction by small molecules (YK-4-279 and TK-216) was shown to repress Ewing sarcoma growth. These molecules are being tested in a phase 1 clinical trial (NCT02657005), whereas their combination with vincristine is currently in phase 2 (NCT05046314). In addition to EWS-FLI1, YK-4-279 was shown to inhibit the function of other oncogenic ETS transcription factors, including ERG and ETV1 [19, 20]. YK-4-279 treatment led to decreased ERG- and ETV1-dependent transcriptional activity, leading to reduced cell motility and invasion of PC cells, and reduced primary tumor growth and metastasis in PC xenografts models [19, 20]. When used in combination with docetaxel, YK-4-279 caused a synergic decrease in PSA levels, suggesting that combined treatments could affect more than one signaling pathway to induce apoptosis and inhibit the growth, migration, and invasion of PC cells [21]. Given these promising results, development of molecules that inhibit the interaction of DHX9 with AR could be similarly therapeutically exploited to dampen AR transcriptional and oncogenic activity in PC.

## Conclusion

Thus, our work uncovers a novel molecular mechanism underlying PC tumorigenic features, which involves DHX9 in the transcriptional program coordinated by AR. Moreover, these findings suggest that the molecular crosstalk between AR and DHX9 could represent a promising target for new therapeutic approaches for PC.

## Supplementary Information

**Additional file 1: Supplementary Fig. 1.** *DHX9* depletion affects tumorigenic phenotype in PC-3 cells. PC-3 cells were transfected with a control siRNA (siCTRL) or a siRNA specific for *DHX9* (si*DHX9*) and *DHX9* downregulation was confirmed by WB analysis (**A**) or by qRT-PCR (**B**). Histograms represent the relative DHX9 expression versus siCTRL. **C** PC-3 cells were transfected as in (**A**) and analyzed by MTS assay after 24, 48 or 72 hrs. Values are the mean ± SD of three independent experiments, each performed in triplicate, considering the siCTRL 24 hrs samples as 1. **D** PC-3 cells were transfected and as in (**A**) for 48 hrs and migration assay was performed. The crystal violet–stained migrating cells were photographed (*left*) and counted (*right*). Values are the mean ± SD of three independent experiments, considering the siCTRL samples as 100. Magnification, × 10. Statistical analysis in (**A**), (**B**), (**C**) and (**D**) were performed by Student’s *t* test, *p* values: *, *p* ≤ 0.05; **, *p* ≤ 0.01; ***, *p* ≤ 0.001. **Supplementary Fig. 2.** *DHX9* depletion affects tumorigenic phenotype in LNCaP cells. **A** LNCaP cells were transfected with either control siRNA (siCTRL) or a siRNA specific for *DHX9* (si*DHX9*) for 48 hrs. *DHX9* downregulation was confirmed by qPCR. Histograms represent the relative *DHX9* RNA expression versus siCTRL, normalized to *GAPDH* expression. **B** Heatmap representing differential gene expression in control (siCTRL) and *DHX9* silencing (si*DHX9*) LNCaP cells. Each column represents a sample, and each row represents a gene. **C** LNCaP cells were transfected as in (**A**) and *DHX9* downregulation was confirmed by WB analysis. Histograms represent the relative DHX9 protein expression versus siCTRL, normalized to GAPDH expression. **D** Gene Ontology (performed using DAVID) of terms regulated at gene expression levels by analyzing DEGs after *DHX9* silencing. Histograms represent the TOP20 Biological Process categories, indicating the Fold Enrichment score and the -log_10_ (*p-*values). Statistical analysis in (**A**) and (**C**) were performed by Student’s *t* test, *p* values: *, *p* ≤ 0.05; **, *p* ≤ 0.01. **E** and (**F**) Gene Ontology (performed using DAVID) of terms regulated at gene expression levels by analyzing upregulated (**E**) and downregulated (**F**) transcripts after *DHX9* silencing. Histograms represent the Fold Enrichment score (in black) and the -log_10_ (*p-*values; in red). **Supplementary Fig. 3.** Androgens modulate *DHX9* expression. **A** The plot shows the Pearson correlation between *AR* and *DHX9* expression in in 370 PCa patients (GSE21034) [25]. LNCaP cells were cultured in medium containing FBS or CSS and the expression of DHX9 was measured by qPCR (**B**) or WB analysis (**C**). Quantification of mRNA and protein level are shown in the bar graphs as the mean ± SD of three independent experiments, considering the control sample (FBS) as 1. Statistical analysis was performed by Student’s *t* test, *p* values: *, *p* ≤ 0.05; ***, *p* ≤ 0.001. **Supplementary Fig. 4.** DHX9 and AR act in a functional complex to modulate gene expression. **A** Total LNCaP extracts were immunoprecipitated with AR antibody (IP AR) and treated or not with RNAse. IP and input were subjected to WB to analyse the expression of DHX9 and AR. **B** Cytosolic, nuclear soluble and nuclear insoluble chromatin-associated fractions of LNCaP cells were analyzed upon DHT treatment by WB, using the indicated antibodies. Histone H3 and GAPDH were evaluated, respectively, as nuclear chromatin associated fraction and cytosolic markers. **C** Pearson correlation analysis on Prostate Cancer patients (GSE21034), performed between the expression of *DHX9* and *TMPRSS2*, *MAOA*, *NDRG1* or *KLK3*. Values are expressed as base 2-logarithm. In each panel, the correlation value (R) and the relative *p-value* are indicated. **D** LNCaP cells were transfected with a control siRNA (siCTRL) or a siRNA specific for *DHX9* (si*DHX9*), cultured for 48 hrs in CSS, and treated or not with DHT (10 nM). qPCR was performed to analyze the *DHX9* expression level. Histogram represents the mean ± SD of three independent experiments, considering the siCTRL CSS sample as 1. Statistical analysis was performed by Student’s *t* test, *p* values: ****, *p* ≤ 0.0001.

**Additional file 2.**

## Data Availability

The datasets deposited during the current study are available at GSE195916.

## Abbreviations

ADT: Androgen-Deprivation Therapy
AR: Androgen Receptor
ChEA: Chromatin Immunoprecipitation Enrichment Analysis
ChIP: Chromatin Immunoprecipitation
CRPC: Castration-Resistant Prostate Cancer
CSS: *Charcoal-Stripped* Serum
DFS: Disease-Free Survival
DHT: 5α*-dihydrotestosterone*
DSB: Double Strand Breaks
EPD: Eukaryote Promoter Database
ETS: E26 transformation-specific
FBS: Fetal Bovine Serum
GEPIA: Gene Expression Profiling Interactive Analysis
KLK3: *Kallikrein 3*
MAOA: Monoamine Oxidase A
NDRG1: N-myc Downstream Regulated Gene 1
PBS: Phosphate Buffered Saline
PC: Prostate Cancer
PSA: Prostate Serum Antigen
RHA: RNA Helicase A
SNAI2: Snail Family Transcriptional Repressor 2
TCGA: The Cancer Genome Atlas
TMPRSS2: Transmembrane Serine Protease 2
