# Supplementary material for: The DNA/RNA helicase DHX9 contributes to the transcriptional program of the androgen receptor in prostate cancer
Source: J Exp Clin Cancer Res. 2022 May 19;41:178. doi: 10.1186/s13046-022-02384-4 (PMC9118622; doi:10.1186/s13046-022-02384-4)

Supplementary Figure 1. *DHX9* depletion affects tumorigenic phenotype in PC-3 cells.

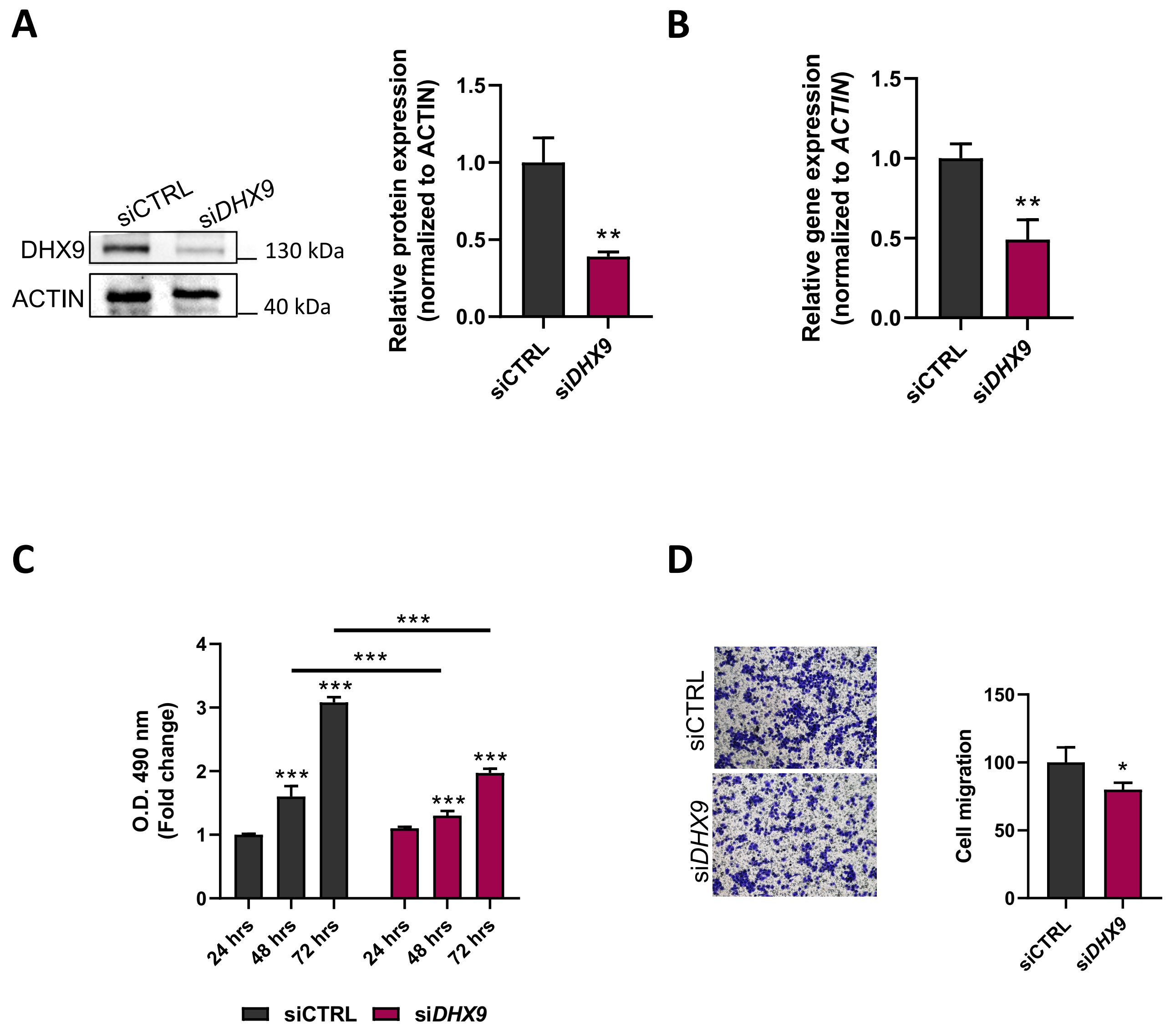

Supplementary Figure 2. *DHX9* depletion affects tumorigenic phenotype in LNCaP cells.

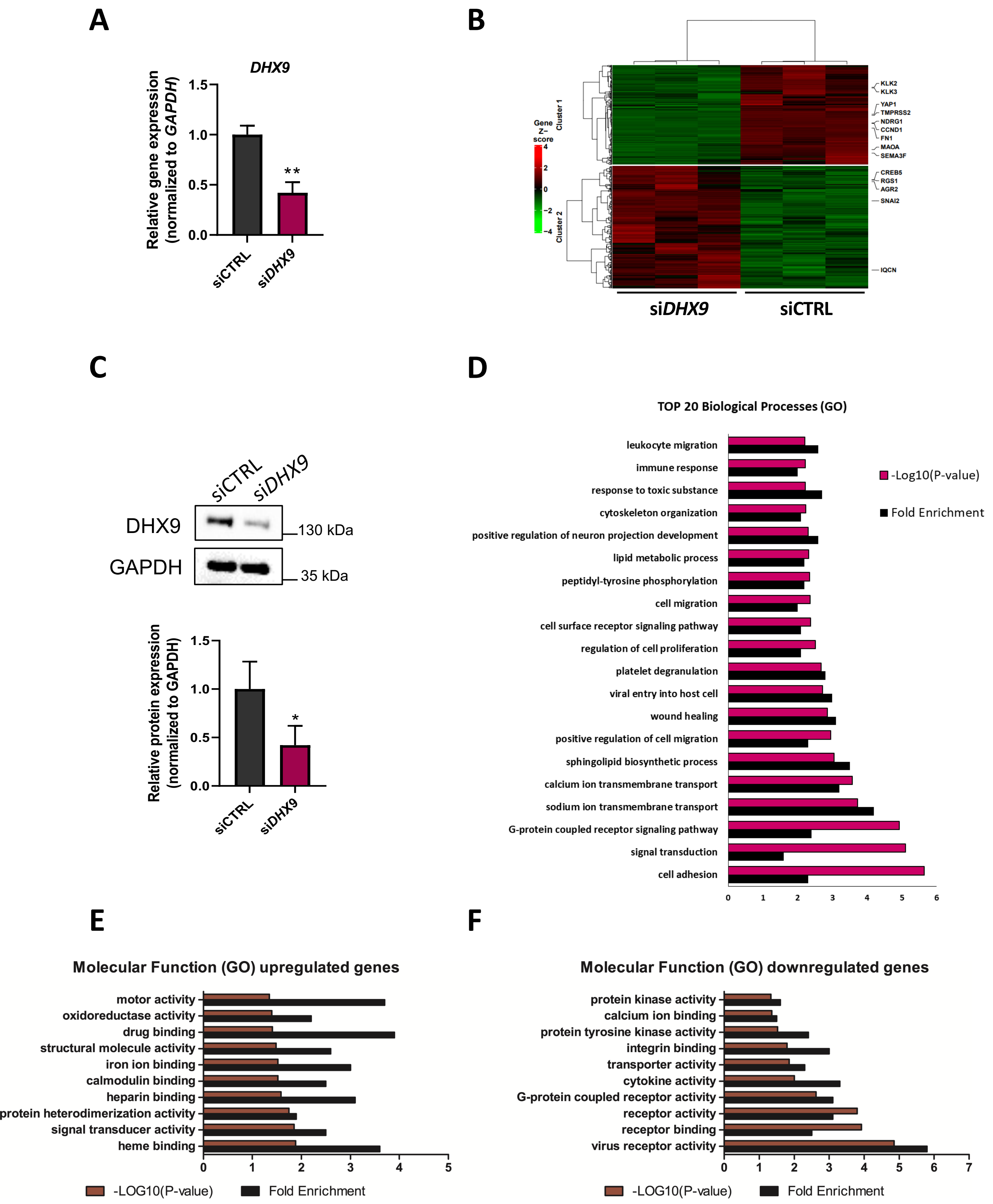

Supplementary Figure 3. Androgens modulate *DHX9* expression.

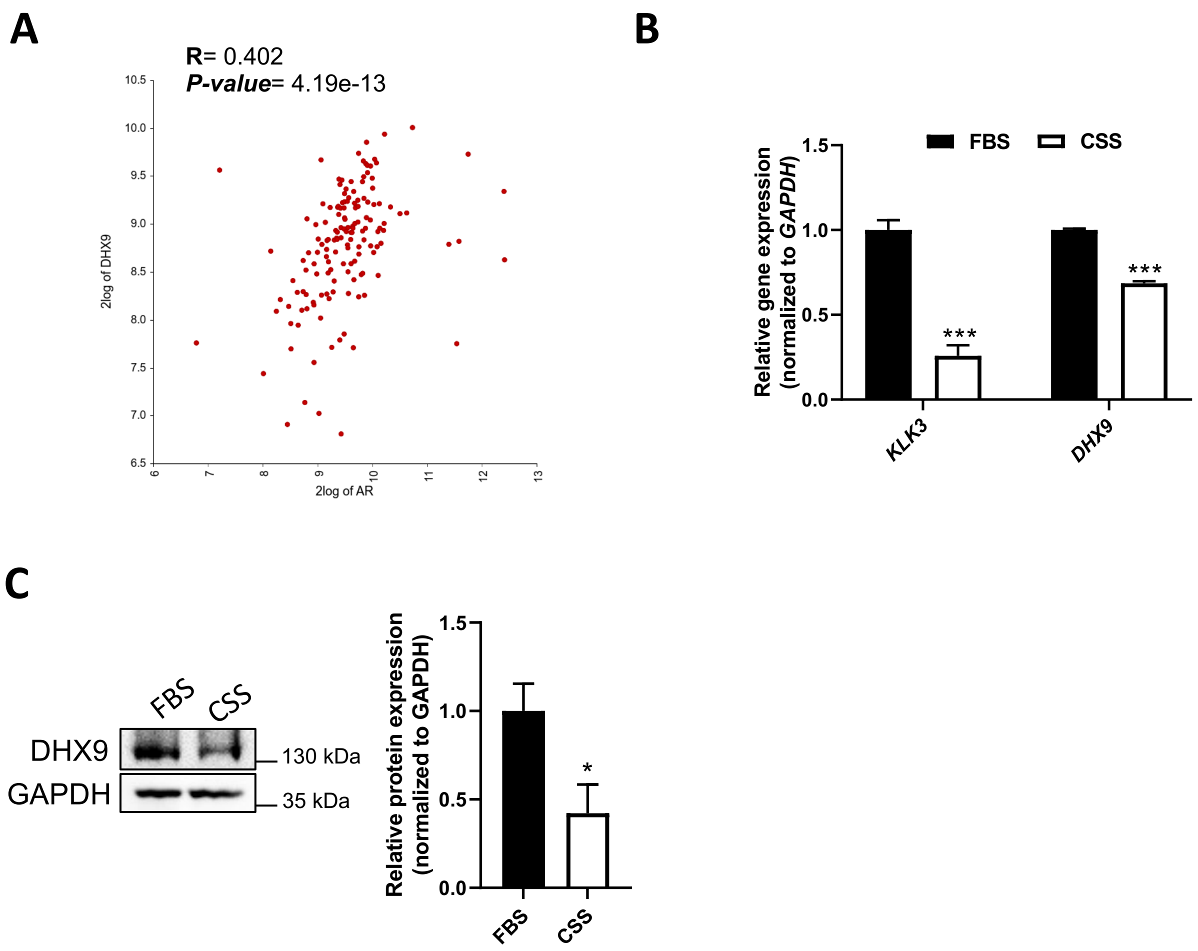

Supplementary Figure 4. DHX9 and AR act in a functional complex to modulate gene expression.

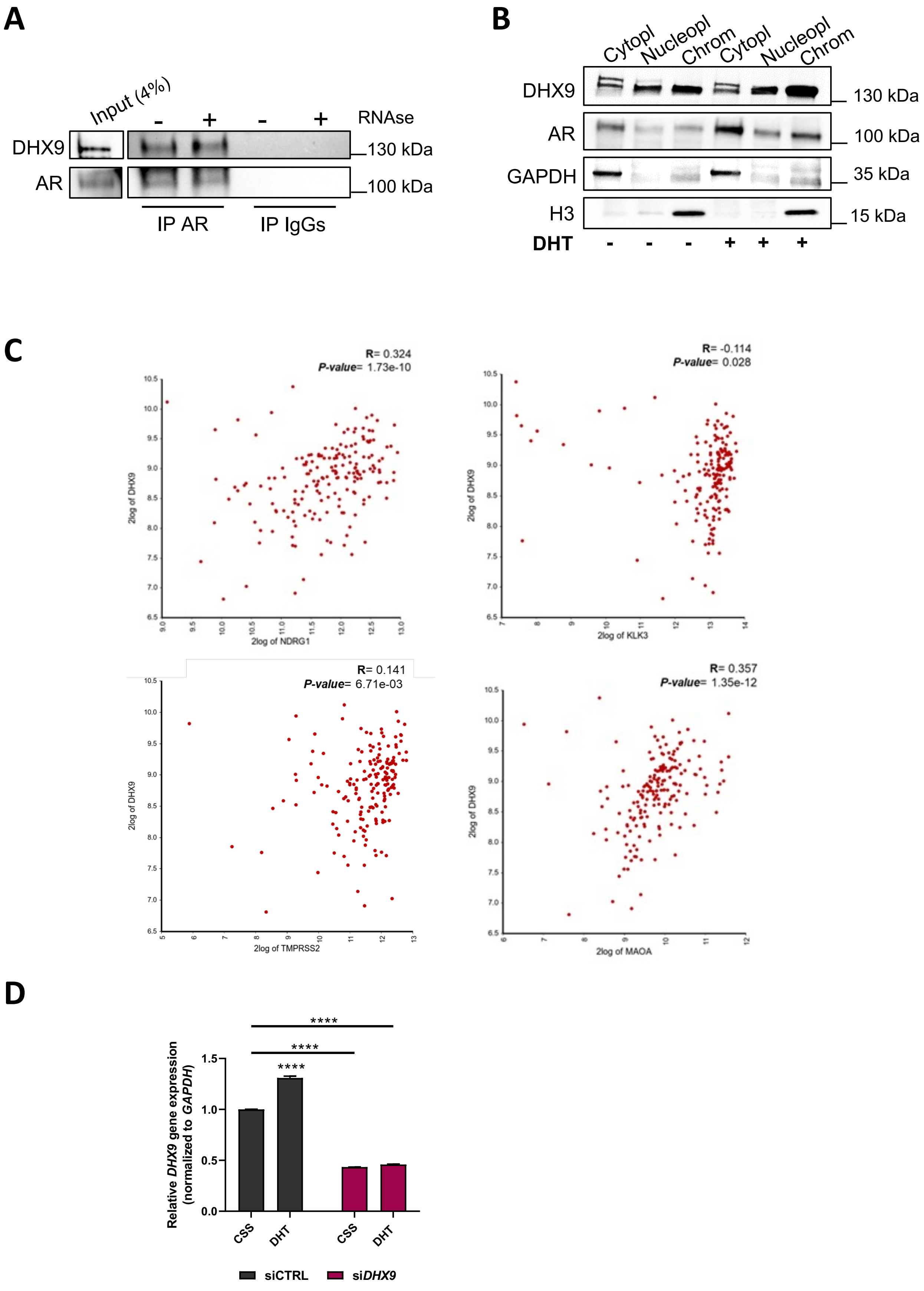

Supplement: Supplementary file 1 — Additional file 1: Supplementary Fig. 1. DHX9 depletion affects tumorigenic phenotype in PC-3 cells. PC-3 cells were transfected with a control siRNA (siCTRL) or a siRNA specific for DHX9 (siDHX9) and DHX9 downregulation was confirmed by WB analysis (A) or by qRT-PCR (B). Histograms represent the relative DHX9 expression versus siCTRL. C PC-3 cells were transfected as in (A) and analyzed by MTS assay after 24, 48 or 72 hrs. Values are the mean ± SD of three independent experiments, each performed in triplicate, considering the siCTRL 24 hrs samples as 1. D PC-3 cells were transfected and as in (A) for 48 hrs and migration assay was performed. The crystal violet–stained migrating cells were photographed (left) and counted (right). Values are the mean ± SD of three independent experiments, considering the siCTRL samples as 100. Magnification, × 10. Statistical analysis in (A), (B), (C) and (D) were performed by Student’s t test, p values: *, p ≤ 0.05; **, p ≤ 0.01; ***, p ≤ 0.001. Supplementary Fig. 2. DHX9 depletion affects tumorigenic phenotype in LNCaP cells. A LNCaP cells were transfected with either control siRNA (siCTRL) or a siRNA specific for DHX9 (siDHX9) for 48 hrs. DHX9 downregulation was confirmed by qPCR. Histograms represent the relative DHX9 RNA expression versus siCTRL, normalized to GAPDH expression. B Heatmap representing differential gene expression in control (siCTRL) and DHX9 silencing (siDHX9) LNCaP cells. Each column represents a sample, and each row represents a gene. C LNCaP cells were transfected as in (A) and DHX9 downregulation was confirmed by WB analysis. Histograms represent the relative DHX9 protein expression versus siCTRL, normalized to GAPDH expression. D Gene Ontology (performed using DAVID) of terms regulated at gene expression levels by analyzing DEGs after DHX9 silencing. Histograms represent the TOP20 Biological Process categories, indicating the Fold Enrichment score and the -log10 (p-values). Statistical analysis in (A) and (C [file 13046_2022_2384_MOESM1_ESM.pdf]
